# Breakfast Dietary Patterns and Their Associations with Metabolic Indicators and NAFLD Severity: A Cross-Sectional Study Among Iranian Adults

**DOI:** 10.34172/hpp.45534

**Published:** 2026-08-21

**Authors:** Mehrdad Kianmehr, Maryam Rafraf, Alireza Hosseini, Mojtaba Kianmehr, Roghayeh Molani-Gol

**Affiliations:** ^1^Student Research Committee, Tabriz University of Medical Sciences, Tabriz, Iran; ^2^Nutrition Research Center, Department of Community Nutrition, Faculty of Nutrition and Food Science, Tabriz University of Medical Sciences, Tabriz, Iran; ^3^Department of Internal Medicine, Faculty of Medicine, Gonabad University of Medical Sciences, Gonabad, Iran; ^4^Department of Medical Physics, Faculty of Medicine, Gonabad University of Medical Sciences, Gonabad, Iran

**Keywords:** Breakfast, Non-alcoholic fatty liver disease, Obesity, Aspartate aminotransferases, Alanine transaminase

## Abstract

**Introduction:**

Non-alcoholic fatty liver disease (NAFLD) is one of the most common chronic diseases worldwide. This study investigated the relationship between breakfast dietary patterns and some metabolic risk factors, obesity, and disease severity in patients with NAFLD.

**Methods:**

This cross-sectional study was conducted on 280 adults with NAFLD. Factor analysis was used to identify the breakfast dietary patterns. Statistical analysis was performed with SPSS 19 software using Linear and Ordinal regression models.

**Results:**

Two "healthy" and "unhealthy" breakfast dietary patterns were identified. Significant inverse relationships were seen between the consumption of the healthy breakfast pattern and total cholesterol (TC) and body mass index (BMI) in the adjusted model. There were significant direct relationships between the consumption of the unhealthy breakfast dietary pattern and alanine aminotransferase (ALT), aspartate aminotransferase (AST), BMI, and waist circumference (WC) after adjustment for confounders. Participants in the third tertile of the unhealthy breakfast pattern had significantly higher TC, triglycerides (TG), fasting blood sugar (FBS), ALT, AST levels, and BMI compared to the lowest tertile following confounder adjustment. Moreover, healthy breakfast pattern consumption was associated with lower NAFLD severity (Adjusted OR: 0.998 (95%CI: 0.997-0.999), *P*=0.029), and unhealthy breakfast pattern consumption was associated with increased severity of NAFLD (Adjusted OR: 1.005 (95%CI: 1.002-1.007), *P*<0.001).

**Conclusion:**

The results suggest that following a healthy breakfast pattern was associated with a better lipid profile and reduced total body adiposity and disease severity, while adherence to an unhealthy breakfast pattern was positively associated with NAFLD risk factors. A healthy breakfast pattern could be considered in the management of NAFLD. Longitudinal or interventional studies are required to establish causality.

## Introduction

 Non-alcoholic fatty liver disease (NAFLD) is one of the most common chronic and non-communicable diseases worldwide, resulting from the accumulation of fat, including triglycerides (TG), in liver cells.^1^ It is a major contributor to liver-related mortality. The global prevalence of this disease is estimated to be 32.4%. This rate is reported to range from 28% to 32.4% in Asia and 38% in Iran.^2^ It has been suggested that NAFLD is a hepatic manifestation of metabolic syndrome that is closely associated with obesity, lipid disorders, insulin resistance, and type 2 diabetes (T2D).^3-5^

 There is no specific drug to treat NAFLD, and to improve it, the underlying risk factors for fatty liver must be eliminated. The most common approach to treatment is to change lifestyle, such as nutrition, eating habits, and physical activity.^6^ Studies conducted on diet have often examined the relationship between one or more nutrients and NAFLD.^7-10^ However, less attention has been paid to the relationship between dietary patterns and disease status and progression. Dietary patterns provide a suitable picture of the diets and nutritional habits of individuals in the community.^11^ The findings of a meta-analysis showed that Western dietary patterns containing high levels of red meat, processed food, refined grains, and high-fat dairy could significantly increase NAFLD.^12^

 In addition to the daily dietary pattern, the breakfast dietary pattern is of particular importance. Breakfast is defined as the first meal of the day, consumed within 2 hours of waking up.^13^ Breakfast is often regarded as the most behaviorally stable meal across populations, with regular consumption patterns observed more consistently than those of lunch or dinner.^14^ Regular breakfast consumption has been linked to improved glucose and lipid metabolism, likely through alignment with endogenous circadian rhythms and diet-induced thermogenesis peak in the earlier part of the day.^15,16^ Breakfast consumption patterns in societies develop over time depending on culture, eating habits, and food availability.^17^ On the other hand, an individual’s physiological status and the presence of various diseases influence food choices.^18^ Some studies have examined breakfast dietary patterns in various diseases, including obesity and metabolic syndrome.^19-21^ In a cross-sectional study on 2086 Swiss adults aged 18 to 75 in 2018, three breakfast dietary patterns were identified, and the “prudent breakfast dietary pattern” (fruits, unprocessed foods, unsweetened whole grain pieces, nuts, seeds, and yogurt) was inversely associated with abdominal obesity.^22^ Moreover, Akbarzadeh et al.^23^ investigated the relationship between breakfast dietary patterns and obesity in 840 healthy Iranian men and women aged 20 to 59 in 2020. They reported that individuals who adhered more to the “sweets, tea, and coffee” and “bread and cereals, meat products, and coffee” breakfast dietary patterns had a higher likelihood of central obesity. Yoo et al.^24^ evaluated the relationship between breakfast consumption patterns and metabolic syndrome using data from 16,734 adults over 18 years old obtained from the National Health and Nutrition Survey of South Korea in 2014. They indicated that among the two identified “traditional Korean” and “dairy-grain” breakfast dietary patterns, the dairy-grain pattern was associated with a reduced risk of metabolic syndrome among breakfast consumers. Regular breakfast consumption has been linked to improved glucose and lipid metabolism, likely through alignment with endogenous circadian rhythms; insulin sensitivity and diet-induced thermogenesis peak in the earlier part of the day. No study is available regarding the relationship between breakfast dietary patterns and NAFLD risk factors.

 Although overall dietary patterns have been linked to NAFLD, there is a consensus that breakfast plays an important role in helping consumers achieve better dietary intake and is also associated with multiple health risk factors.^25,26^ However, limited evidence exists regarding the specific contribution of breakfast dietary habits in NAFLD. We hypothesized that adherence to a healthy breakfast pattern would be inversely associated with metabolic abnormalities and NAFLD severity. Therefore, the present study aimed to investigate breakfast dietary patterns and their association with some metabolic factors, including lipid profile, fasting blood sugar (FBS), and liver enzymes, anthropometric indices, and disease severity in adults with NAFLD in Gonabad city, Khorasan Razavi province, Iran.

## Materials and methods

###  Study population and design

 The present cross-sectional study was conducted on 280 individuals (males and females) aged 18-65 years with newly diagnosed NAFLD living in Gonabad city, Iran, from June to December 2024. All procedures were conducted following the Declaration of Helsinki. The selection of patients was done through a gastroenterologist referral using the convenience sampling method due to its cost-effectiveness, less time-consuming, and simple operation. Because the minimum recommended sample size for factor analysis is between 3 and 20 times the number of variables,^27^ and because we did not know in advance how many food groups would remain after the factor analysis, we initially targeted 10 participants per predefined food group (28 groups in Supplementary Table 1), yielding a total sample size of 280. Following data screening and initial factor analysis, 15 groups were merged or excluded due to overlapping loadings and insufficient variance, resulting in 13 food groups retained for the final factor analysis. The sample size was considered adequate based on the observed factor structure and fit indices of the model, which supported stable estimation of dietary pattern structures in exploratory factor analysis. The study process is depicted in Figure 1.

**Figure 1:**
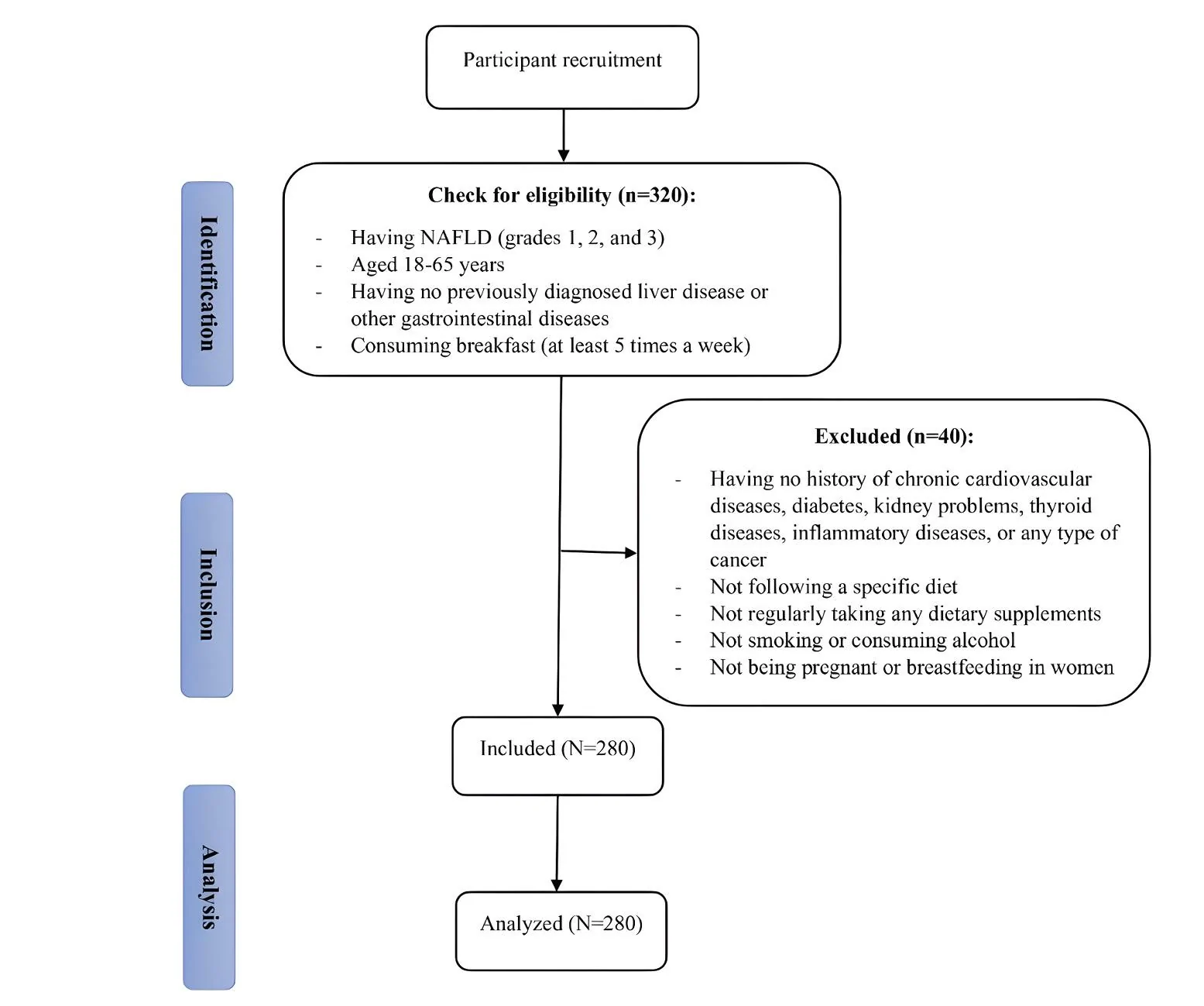
flowchart of the study sample, NAFLD: Non-alcoholic fatty liver disease

 The inclusion criteria for the study were: individuals with NAFLD (grades 1, 2, and 3), aged between 18 and 65 years, have no previously diagnosed liver disease or other gastrointestinal diseases, and consuming breakfast (at least 5 times a week). The exclusion criteria were: no history of chronic cardiovascular diseases, diabetes, kidney problems, thyroid diseases, inflammatory diseases, or any types of cancer, not following a specific diet, not regularly taking any dietary supplements, not smoking or consuming alcohol, not being pregnant or breastfeeding in women, and not being a shift worker. All participants or their legal guardians were informed about the study process before completing the questionnaire and signing a written informed consent form. This study procedure was approved by the Ethics Committee of Tabriz University of Medical Sciences (Ethics Code: IR.TBZMED.REC.1402.750). Moreover, the Research Vice-Chancellor of Tabriz University of Medical Sciences, Tabriz, Iran, registered the protocol of this study (grant number: 73108).

###  Liver ultrasonography and definitions of NAFLD

 Liver ultrasound examinations were performed using the RS80A ultrasound system (Samsung, South Korea). NAFLD diagnosis was based on the American Gastroenterological Association recommendations. NAFLD was diagnosed when ultrasound examinations revealed hepatic steatosis at any stage, with or without associated biochemical changes.^28^

 The grading of NAFLD was performed by an experienced radiologist using the B-Mode Ultrasound method. To avoid bias, the radiologist was blinded to the participants’ laboratory and clinical details at the time of the procedure. NAFLD was defined as markedly increased echogenicity of the body, weakly in the posterior part of the right lobe of the body, and no or poor visualization of the patient and the diaphragm. Grade 0: Normal, Grade 1: Mild steatosis that is moderately increased in echogenicity of the device compared with normal renal walls and diaphragm. Grade 2: Severe steatosis that is severely diffused in echogenicity of the device compared with renal walls, with poor visualization of the renal walls. Grade 3: Markedly increased in echogenicity of the liver with poor visualization of the deep parenchyma of the body and diaphragm.^29^

###  Data collection

 Demographic characteristics, including age, marital status, gender, education, and occupation, were collected using a questionnaire.

###  Anthropometric measures

 The body weight was measured by a Seca scale (Seca, Hamburg, Germany) without shoes and in minimal clothing, nearest to 0.1 kg. Height was also recorded by wall-mounted tape without shoes, nearest to 0.5 cm. Body Mass Index (BMI) was calculated as the weight (kg) divided by the height (m^2^). Waist circumference (WC) was measured at the midpoint between the lowest rib and the superior border of the iliac crest using a tape and was approximately recorded at 0.5 cm.^30^

###  Physical activity levels

 The physical activity level of study participants was estimated by the International Physical Activity Questionnaire Short Form (IPAQ-SF).^31^ The subjects were categorized as possessing vigorous, moderate, or light physical activitylevels based on the categorical scoring guidelines of the IPAQ-SF.^32^

###  Biochemical parameters

 Blood samples were obtained from each subject after 10-12 hours of fasting. The serum was separated by centrifugation at 2000 rpm for 15 minutes and stored at –70 °C until analysis. The measurement of FBS, TG, total cholesterol (TC), and high-density lipoprotein cholesterol (HDL-C) was performed by the enzymatic colorimetric method using enzymatic colorimetric kits (Pars Azmoon, Tehran, Iran). The Friedewald formula was applied for low-density lipoprotein cholesterol (LDL-C) calculation: LDL-C = TC (mg/dl) _ ([HDL-C (mg/dl) + TG (mg/dl)]/5).^33^ The normal reference ranges for these measures were defined as: FBS 70–99 mg/dL, TC < 200 mg/dL, TG < 150 mg/dL, HDL-C > 40 mg/dL for men and > 50 mg/dL for women, and LDL-C < 100 mg/dL.

 Measurement of alanine aminotransferase (ALT) and aspartate aminotransferase (AST) enzyme activity was based on the International Federation of Clinical Chemistry (IFCC) method, and the reference range for them was: ALT 7–40 U/L and AST 9–40 U/L.^34^

###  Dietary intakes

 The dietary intakes of subjects were evaluated by the 24-hour dietary recall method for three days (two weekdays and one weekend day). Given that the breakfast consumption patterns and timing are generally more behaviorally stable than other meals, a 3-day recall period was considered adequate for capturing usual breakfast habits. Trained interviewers collected dietary recalls using a standardized multiple-pass method; the first recall was face-to-face during the participants’ first visit, and others were conducted by phone interview. This approach was selected to reduce intra-individual day-to-day variability and to provide a more reliable estimate of habitual intake compared with a single recall. Participants were asked to report the types and amounts of foods consumed in the past 24 hours. Portion sizes were estimated using household measures and food models to enhance accuracy. Consequently, consumed food portion sizes were converted to grams by household measures.^35^ Finally, participants’ diets were assessed using Nutritionist IV software (First Databank, San Bruno, CA, USA) modified for Iranian foods to obtain the participants’ energy intake at breakfast and daily energy intakes.

###  Definition of breakfast composition

 Breakfast was defined as the first meal of the day eaten within two hours of waking up, typically no later than 10:00 AM.^36^ To identify the breakfast dietary patterns, factor analysis was used. Food items were divided into 28 food groups based on their composition and content, and previous studies.^37,38^ In the next step, the KMO index and Bartlett test were used to determine the model’s suitability. To identify the breakfast dietary patterns based on the exploratory factor analysis method, after calculating the correlation matrix between the variables, groups were extracted by the main axes classification method, in which variables with a high correlation are placed in one group. Thus, to identify the breakfast dietary patterns, a Maximum Likelihood Analysis with Varimax rotation was performed on 28 food groups. To determine the number of factors (breakfast dietary patterns), the change point in the behavior of the scree plot curve was used. Finally, two breakfast dietary patterns were considered. According to previous studies, values with a factor loading greater than or equal to 0.2 were considered to determine the items of each dietary pattern. The naming of the factors was performed collaboratively by a nutritionist and a gastroenterologist based on the interpretation of the food items in each factor. Each individual was given a score based on the amount of food consumed in different groups and their factor loading for each breakfast dietary pattern. Then, based on the tertiles of the breakfast dietary patterns, the scores were classified as low, medium, and high. Supplementary Table 1 shows the food groups used in the analysis of breakfast dietary patterns.

###  Statistical analysis

 Data were analyzed using the SPSS software (SPSS Inc., Chicago, Version 19). Linear regression analysis was conducted in both crude and adjusted models to examine the relationship between the scores of each breakfast dietary pattern and other study variables. Quantitative variables were entered into the model as they were, while one of the subgroups was considered as a reference for qualitative variables. Gender as a nominal variable (male as reference), severity of NAFLD as an ordinal variable (grade 1, 2, and 3), and breakfast dietary patterns tertiles as an ordinal variable (T1, T2, and T3) were entered. Also, age, weight, BMI, WC, daily energy intake, breakfast dietary pattern score, physical activity, and all blood test parameters were entered as quantitative variables in the regressions. To assess the normality of the residuals in the linear regression model, the Kolmogorov-Smirnov test was used, which indicated that the residuals follow a normal distribution. To investigate the relationship between NAFLD grade and breakfast dietary patterns along with control variables, the ordinal regression was applied in both crude and adjusted models, and the odds ratio for the NAFLD severity in healthy and unhealthy breakfast dietary patterns was determined with a 95% confidence interval (CI). In both methods, daily energy intake, physical activity level, gender, and age were included as confounding variables. A* p-*value less than 0.05 was considered significant.

## Results

 The general characteristics of the participants are presented in Table 1. Two hundred eighty patients with NAFLD participated in this study. The mean age of participants was 46.06 (SD = 12.37) years, and 63.6% and 36.4% were female and male, respectively. Moreover, 92.5% of patients were married, and 72.1% were employed. The mean BMI was 29.19 (SD = 4.14) kg/m^2^, and the mean daily breakfast energy intake was 710.98 (SD = 254.52) kcal. In terms of physical activity levels, 63.2%, 30.7%, and 6.1% had Light, moderate, and vigorous levels of physical activity, respectively. Regarding the disease severity, 24.6%, 63.2%, and 12.2% of participants had grades 1, 2, and 3 NAFLD, respectively.

**Table 1:** Characteristics of the study participants (n = 280)

**Characteristics **	**Mean±SD or n (%)**
Age (year)	46.06 ± 12.37
Sex n (%) Male	102 (36.4)
Marital status Married n (%)	259 (92.5)
Education n (%) Illiterate Primary education High school education Higher education	10 (3.6) 38 (13.5) 100 (35.7) 132 (47.1)
Job n (%) Employed	202 (72.1)
Weight (kg)	81.61 ± 13.84
Height (cm)	167.12 ± 9.89
BMI (kg/m^2^)	29.19 ± 4.14
WC (cm)	100.99 ± 9.24
Daily energy intake (kcal)	2899.61 ± 783.33
Daily breakfast energy intake (kcal)	710.98 ± 254.52
Physical activity (MET-min/week)	761.63 ± 1329.36
PAL n (%) Light Moderate Vigorous	177 (63.2) 86 (30.7) 17 (6.1)
TG (mg/dl)	186.32 ± 84.66
TC (mg/dl)	193.97 ± 37.65
LDL-C (mg/dl)	113.74 ± 30.02
HDL-C (mg/dl)	42.12 ± 11.68
FBS (mg/dl)	95.35 ± 8.71
ALT (U/L)	54.69 ± 32.35
AST (U/L)	37.19 ± 19.06
NAFLD grade n (%) 1 2 3	69 (24.6) 177 (63.2) 34 (12.2)

BMI: Body mass index, WC: Waist circumference, PAL: Physical activity level, TG: Triglycerides, TC: Total Cholesterol, LDL-C: Low-Density Lipoprotein cholesterol, HDL-C: High-Density Lipoprotein cholesterol, FBS: Fasting blood sugar, ALT: Alanine Aminotransferase, AST: Aspartate Aminotransferase, NAFLD: Non-alcoholic fatty liver disease

 As shown in Table 2, two breakfast dietary patterns were obtained. The highest factor loading in the unhealthy breakfast dietary pattern was related to the red meat food group (0.998), and in the healthy breakfast dietary pattern was for the vegetable food group (0.833). The lowest factor loading in the unhealthy breakfast dietary pattern was for the sweets food group (0.248), and in the healthy breakfast dietary pattern, it was connected to the tea and coffee food group (0.202). The KMO index is 0.51, indicating that the data are sufficiently adequate. Additionally, Bartlett’s test was also significant (*P* < 0.001), confirming the suitability of the data for factor analysis. Food groups with a loading factor higher than 0.2 were considered to be important contributors to the dietary pattern.

**Table 2:** Factor loadings for each of the identified breakfast dietary patterns*

**Food Group**	**Food items**	**Breakfast dietary patterns**	
		**Healthy pattern**	**Unhealthy pattern**
Vegetables	Lettuce, spinach, greens, dark greens, parsley, watercress, coriander, dill, leek, mint, basil, grape leaves, beet leaves, onions, cucumbers, chives, radishes, turnips, rhubarb, artichokes, Eggplant, pumpkin, celery, pepper, bell pepper, beet, shallot, garlic, mushroom, okra, green peas, beans, green beans, carrots, pumpkin pudding	0.833	-
Tomato	Tomatoes, tomato juice, tomato paste, ketchup	0.679	-
Low-fat dairy products	Low-fat milk, Low-fat cocoa milk, low-fat yogurt, low-fat cheese, pasteurized curd, buttermilk, Qarat	0.240	-
Whole grains	dark breads (barbari, sangak, taftun), barley bread, wheat, sprouts, corn	0.208	-
Nuts	Chickpeas, walnuts, hazelnuts, almonds, pistachios, peanuts, cashews, sesame, seeds	0.208	-
Tea and coffee	Tea and coffee	0.202	-
Red meat	Beef, lamb, camel meat, hamburger	-	0.998
Soft drinks and industrial juices	Industrial fruit juices with added sugar, soft drinks, syrups, malt beer	-	0.558
Mayonnaise	Types of mayonnaise and salad dressings	-	0.350
Animal oil and butter	Cow oil, sheep oil, industrial butter, local butter, tail, milks fat	-	0.282
Full-fat dairy products	Full-fat milk, full-fat yogurt, full-fat cheese	-	0.269
Refined grains	White breads (lavash, baguettes), ice cream bread, starch, wheat flour, rice, pasta, noodles,	-	0.267
Sweets	Cake, cookie, candies, dumpling, gaz, sohaan, noghl, Danish, sugar cubes, sugar, chocolate, pastille, jelly, caramel cream, chocolate biscuit, all kinds of jams and compotes, biscuit with cream	-	0.248

The KMO index is 0.512, indicating that the data is sufficiently adequate, and the significance of the Bartlett’s test also shows that there is a sufficient correlation between the food groups (χ2 = 713.14 and *P* < 0.001) eigenvalue > 1 *Factor loadings of < 0.2 were not reported Factor variance for healthy and unhealthy dietary breakfast patterns was 14.25% and 17.21%, respectively

 The association of breakfast dietary patterns with biochemical parameters, anthropometric measures, and NAFLD grade is shown in Table 3. There was a statistically significant inverse association between the consumption of the healthy breakfast dietary pattern with serum TC (*P* = 0.032), and BMI (*P* = 0.041) after adjustment for age, gender, physical activity level, and total daily energy intake. Furthermore, an unhealthy breakfast dietary pattern had a statistically significant positive association with ALT (*P* < 0.001), AST (*P* < 0.001), BMI (*P* < 0.001), and WC (*P* = 0.038) in the adjusted model. Unhealthy breakfast dietary patterns had no significant association with the lipid profile parameters in both crude and adjusted models (*P* > 0.05). In addition, there was no statistically significant relationship between healthy and unhealthy breakfast dietary patterns with serum FBS and weight in the crude model and following adjustment for confounders (*P* > 0.05).

**Table 3:** Association of breakfast patterns with metabolic factors and NAFLD grade (n = 280)

**Variable**	**Healthy breakfast dietary pattern**				**Unhealthy breakfast dietary pattern**			
	**Crud model**		**Adjusted model**^a^		**Crud model**		**Adjusted model**^a^	
	**B (CI)**	***P*** **-value**	**B (CI)**	***P*** **-value**	**B (CI)**	***P*** **-value**	**B (CI)**	***P*** **-value**
TG (mg/dl)	-0.031 (-0.091 – 0.028)	0.303	-0.020 (-0.082 – 0.041)	0.514	0.028 (-0.069 – 0.126)	0.569	0.008 (-0.093 – 0.108)	0.883
TC (mg/dl)	-0.028 (-0.055 – -0.002)	0.037	-0.030 (-0.057 – -0.003)	0.032	0.026 (-0.017 – 0.070)	0.230	0.033 (-0.011 – 0.078)	0.138
LDL-C (mg/dl)	-0.009 (-0.030 – 0.012)	0.389	-0.008 (-0.030 – 0.014)	0.460	0.006 (-0.029 – 0.041)	0.736	0.001 (-0.035 – 0.037)	0.954
HDL-C (mg/dl)	0.009 (0.001 – 0.018)	0.025	0.008 (-0.0002 – 0.017)	0.061	-0.0003 (-0.014 – 0.013)	0.968	0.004 (-0.010 – 0.018)	0.552
FBS (mg/dl)	-0.002 (-0.004 – 0.010)	0.182	-0.003 (-0.004 – 0.010)	0.266	-0.001 (-0.009 – 0.011)	0.903	-0.001 (-0.009 – 0.011)	0.804
ALT (U/L)	-0.003 (-0.025 – 0.020)	0.826	-0.001 (-0.024 – 0.022)	0.938	0.044 (0.007 – 0.081)	0.020	0.053 (0.015 – 0.090)	0.006
AST (U/L)	-0.003 (-0.011 – 0.016)	0.696	-0.001 (-0.013 – 0.015)	0.869	0.039 (0.018 – 0.061)	< 0.001	0.042 (0.019 – 0.064)	< 0.001
Weight (kg)	0.007 (-0.003 – 0.017)	0.169	-0.003 (-0.006 – 0.013)	0.495	0.012 (-0.004 – 0.028)	0.150	0.008 (-0.008 – 0.024)	0.344
BMI (kg/m^2^)	-0.001 (-0.004 – 0.002)	0.547	-0.002 (-0.004 – -0.001)	0.041	0.007 (0.002 – 0.012)	0.003	0.009 (0.005 – 0.014)	< 0.001
WC (cm)	-0.005 (-0.012 – 0.001)	0.098	-0.002 (-0.009 – 0.004)	0.520	0.013 (0.002 – 0.024)	0.016	0.011 (0.001 – 0.022)	0.038
NAFLD grade	0.999* (0.997-1.001)	0.128	0.998* (0.997-0.999)	0.029	1.004* (1.002-1.007)	0.001	1.005* (1.002-1.007)	< 0.001

B: B Coefficient, CI: Confidential Interval, TG: Triglycerides, TC: Total Cholesterol, LDL-C: Low-Density Lipoprotein cholesterol, HDL-C: High-Density Lipoprotein cholesterol, FBS: Fasting blood sugar, ALT: Alanine Aminotransferase, AST: Aspartate Aminotransferase, BMI: Body Mass Index, WC: Waist circumference, Non-alcoholic fatty liver disease The data were analyzed using linear regression *Odds Ratio (OR) that was analyzed using ordinal regression ^a^Adjusted for age, gender, physical activity level, and total daily energy intake The *P*-value of less than 0.05 is statistically significant

 The odds of being in a higher grade of NAFLD decreased by 0.2% for each unit increase in the healthy breakfast pattern score (*P* = 0.029) after adjusting for age, gender, physical activity level, and total daily energy intake. Moreover, the odds of being in a higher grade of NAFLD increased by 0.5 % for each unit increase in the unhealthy breakfast pattern score in the adjusted model (*P* < 0.001).

 The association of breakfast patterns tertiles with biochemical parameters, anthropometric measures, and NAFLD grade is presented in Table 4. There was a statistically significant inverse relationship between the third tertile of the healthy breakfast dietary pattern in the adjusted model with TC (B Coefficient (B) = -16.28, *P* = 0.029). No significant relationship was found between other biochemical parameters and the tertiles of healthy breakfast dietary pattern in the crude and adjusted models (*P* > 0.05).

**Table 4:** Association of breakfast dietary patterns tertiles with metabolic factors and NAFLD grade (n = 280)

**Variable**	**Healthy breakfast dietary pattern**					**Unhealthy breakfast dietary pattern**				
	**T1**	**T2**		**T3**		**T1**	**T2**		**T3**	
		**B (CI)**	***P*** **-value**	**B (CI)**	***P*** **-value**		**B (CI)**	***P*** **-value**	**B (CI)**	***P*** **-value**
TG (mg/dl)	Ref	-12.07 (-45.07–20.93) -15.82 (-48.52–16.88)	0.472^a^ 0.342^b^	-10.87 (-21.92–43.65) -4.41 (-28.58–37.42)	0.515^a^ 0.792^b^	Ref	8.86 (-32.48–14.75) 12.29 (-11.02–35.62)	0.461^a^ 0.300^b^	50.57 (7.60–93.53) 47.89 (2.56–93.22)	0.021^a^ 0.038^b^
TC (mg/dl)	Ref	-7.84 (-22.54–6.86) -10.29 (-24.78–4.20)	0.295^a^ 0.163^b^	-13.20 (-27.81–1.40) -16.28 (-30.91– -1.66)	0.076^a^ 0.029^b^	Ref	4.95 (-15.47–5.57) 6.07 (-4.24–16.40)	0.355^a^ 0.247^b^	19.22 (0.07–38.36) 24.49 (4.43–44.56)	0.049^a^ 0.017^b^
LDL-C (mg/dl)	Ref	-2.66 (-14.46–9.12) -1.34 (-13.07–10.37)	0.656^a^ 0.821^b^	-1.57 (-13.29–10.14) -0.42 (-12.29–11.40)	0.792^a^ 0.944^b^	Ref	0.81 (-7.66–9.28) 1.60 (-6.79–10.003)	0.851^a^ 0.707^b^	1.98 (-13.43–17.39) 0.54 (-16.86–15.78)	0.800^a^ 0.948^b^
HDL-C (mg/dl)	Ref	2.006 (-6.57–2.55) 1.85 (-6.38–2.66)	0.388^a^ 0.420^b^	0.71 (-3.81–5.25) 0.53 (-4.03–5.10)	0.755^a^ 0.816^b^	Ref	-0.17 (-3.46–3.12) -0.25 (-3.50–3.001)	0.919^a^ 0.878^b^	-0.70 (-6.70–5.29) -2.96 (-9.28–3.36)	0.817^a^ 0.357^b^
FBS (mg/dl)	Ref	-2.35 (-5.76–1.06) -2.21 (-5.52–1.09)	0.176^a^ 0.188^b^	-1.32 (-4.71–2.06) -0.78 (-4.12–2.55)	0.441^a^ 0.643^b^	Ref	4.68 (2.33–7.02) 4.04 (1.78–6.31)	< 0.001^a^ 0.001^b^	6.29 (2.02–10.56) 8.02 (3.62–12.41)	0.004^a^ < 0.001^b^
ALT (U/L)	Ref	7.30 (-5.36–19.98) 6.99 (-5.44–19.43)	0.257^a^ 0.269^b^	2.79 (-9.79–15.38) 2.96 (-9.58–15.51)	0.662^a^ 0.642^b^	Ref	-3.79 (-12.27–4.69) -4.68 (-12.70–3.33)	0.380^a^ 0.251^b^	49.97 (34.53–65.41) 61.65 (46.05–77.25)	< 0.001^a^ < 0.001^b^
AST (U/L)	Ref	1.58 (-5.91–9.07) 0.94 (-6.16–8.41)	0.678^a^ 0.805^b^	0.93 (-6.51–8.37) 0.04 (-7.49–7.59)	0.806^a^ 0.990^b^	Ref	-0.39 (-5.22–4.44) -0.69 (-5.39–4.007)	0.874^a^ 0.772^b^	35.88 (27.09–44.68) 41.24 (32.11–50.38)	< 0.001^a^ < 0.001^b^
Weight (kg)	Ref	-2.65 (-8.06–2.57) -3.31 (-8.51–1.88)	0.335^a^ 0.211^b^	0.20 (-5.17–5.57) -0.84 (-6.09–4.40)	0.941^a^ 0.751^b^	Ref	3.82 (-0.04–7.70) 3.45 (-0.26–3.57)	0.053^a^ 0.069^b^	4.48 (-2.55–11.53) 3.38 (-3.84–10.01)	0.211^a^ 0.357^b^
BMI (kg/m^2^)	Ref	-1.29 (-3.53– -0.31) -2.32 (-3.85– -0.80)	0.020^a^ 0.003^b^	-1.18 (-2.78–0.41) -1.44 (-2.98–0.09)	0.145^a^ 0.066^b^	Ref	1.90 (0.76–3.04) 1.94 (0.86–3.02)	0.001^a^ < 0.001^b^	1.78 (-0.29–3.86) 1.91 (0.81–3.01)	0.076^a^ 0.007^b^
WC (cm)	Ref	-2.92 (-6.53–0.87) -3.61 (-7.12– -0.10)	0.111^a^ 0.044^b^	-0.83 (-4.41–2.75) -1.64 (-5.18–1.90)	0.648^a^ 0.362^b^	Ref	1.87 (-0.70–4.46) 2.34 (-0.17–4.85)	0.154^a^ 0.068^b^	4.71 (0.01–9.41) 4.28 (-0.60–9.01)	0.049^a^ 0.085^b^
NAFLD grade	Ref	0.267^*^ (0.118–0.604) 0.204^*^ (0.088–0.469)	0.002^a^ < 0.001^b^	0.218^*^ (0.096–0.494) 0.160^*^ (0.068–0.376)	< 0.001^a^ < 0.001^b^	Ref	0.838^*^ (0.670–2.121) 1.150^*^ (0.635–2.081)	0.550^a^ 0.644^b^	8.101^*^ (2.860–22.942) 13.776^*^ (4.318–43.900)	< 0.001^a^ < 0.001^b^

B: B Coefficient, CI: Confidential Interval, TG: Triglycerides, TC: Total Cholesterol, LDL-C: Low-Density Lipoprotein cholesterol, HDL-C: High-Density Lipoprotein cholesterol, FBS: Fasting blood sugar, ALT: Alanine Aminotransferase, AST: Aspartate Aminotransferase, BMI: Body Mass Index, WC: Waist circumference, Non-alcoholic fatty liver disease The data were analyzed using linear regression *Odds Ratio (OR) that was analyzed using ordinal regression ^a^Crud model ^b^ Adjusted for age, gender, physical activity level, and total daily energy intake The *P*-value of less than 0.05 is statistically significant

 There was a statistically significant inverse relationship between the second tertile of the healthy breakfast dietary pattern with BMI (B = -2.32, *P* = 0.003) and WC (B = -3.61, *P* = 0.044) in the adjusted model. In addition, no significant relationship was seen between the tertiles of healthy breakfast dietary patterns and weight in both crude and adjusted models (*P* > 0.05).

 A statistically significant positive relationship was found between both the second and third tertiles of the unhealthy breakfast dietary pattern and serum FBS levels (*P* = 0.001 and *P* < 0.001, respectively) after adjustment for confounders. There was also a statistically significant positive relationship between the third tertile of the unhealthy breakfast dietary pattern in the adjusted model with TC (B = 24.49, *P* = 0.017), TG (B = 47.89, *P* = 0.038), ALT (B = 61.65, *P* < 0.001), and AST (B = 41.24, *P* < 0.001). The results were not significant for the relationship between the second and third tertiles of the unhealthy breakfast dietary pattern with other biochemical parameters in the crude and adjusted models (*P* > 0.05). A significant positive relationship was found between BMI and both the second (B = 1.94, *P* < 0.001) and third (B = 1.91, *P* = 0.007) tertiles of the unhealthy breakfast dietary pattern in the adjusted model. Also, in the crude model, a significant positive relationship was observed between WC and the third tertile of the unhealthy breakfast dietary pattern (*P* = 0.049). No significant relationship was found between the tertiles of unhealthy breakfast dietary patterns and weight in the crude and adjusted models (*P* > 0.05).

 After adjustment for age, gender, physical activity level, and total daily energy intake, adherence to the second (OR: 0.204, 95% CI (0.088–0.469), *P* < 0.001) and third (OR: 0.160, 95% CI (0.068–0.376), *P* < 0.001) tertiles of the healthy breakfast dietary pattern was associated with a 79.6% and 84% decrease in the odds of NAFLD grade. Moreover, the odds of enhancing the grade of NAFLD were 13.77 times higher in the third tertile of unhealthy breakfast dietary pattern than in the first tertile after adjusting for confounders (OR: 13.776, 95% CI (4.318–43.900), *P* < 0.001).

 Overall, adherence to a healthy breakfast dietary pattern was associated with reduced total cholesterol levels, BMI, and NAFLD severity, whereas an unhealthy breakfast dietary pattern was associated with increased serum levels of liver enzymes, fasting blood glucose, total cholesterol, triglycerides, and BMI, waist circumference, and NAFLD severity in adults with NAFLD.

## Discussion

 This cross-sectional study was the first study to evaluate the association of breakfast dietary patterns with metabolic risk factors, obesity indices, and disease severity in NAFLD patients. In the current study, daily breakfast energy intake was 24.51% of total daily energy intake. Based on the findings, two breakfast dietary patterns were identified, including healthy and unhealthy breakfast dietary patterns by factor analysis. The healthy breakfast dietary pattern included vegetables, tomatoes, low-fat dairy products, whole grains, nuts, tea, and coffee, and the unhealthy breakfast dietary pattern included red meat, soda and industrial fruit juices, mayonnaise, animal fat and butter, high-fat dairy products, refined grains, and sweets.

 NAFLD has been shown to be associated with atherogenic dyslipidemia,^39^ which was also present in our study subjects. Based on the results, greater adherence to the healthy breakfast dietary pattern was associated with decreased serum TC levels. Although there was no significant relationship between the unhealthy breakfast dietary pattern and lipid profile, higher consumption of the unhealthy breakfast, which includes the third of the unhealthy breakfast dietary pattern, was significantly associated with increased TC and TG levels. These results were similar in part to findings of some previous studies, which evaluated dietary patterns in patients with NAFLD or other diseases.^38,40,41^ A meta-analysis study by Del Bo et al.^42^ in adults with NAFLD showed that the Mediterranean dietary pattern (including high consumption of fruits, vegetables, whole grains, legumes, seeds and nuts, and olive oil) can reduce plasma TC. In a study by Sofía Montemayor et al.^43^ on adults with metabolic syndrome, the consumption of a Mediterranean dietary pattern for 12 weeks was effective in increasing HDL-C. A study by Min et al.^41^ on adults with metabolic syndrome revealed that a breakfast pattern of “rice, kimchi, and vegetables” and another breakfast pattern of “eggs, bread, and processed meat” were positively associated with TG in women and men, respectively.

 The studies mentioned above indicates that greater adherence to a dietary pattern containing vegetables, whole grains, nuts, and low-fat dairy products are accompanied with a better lipid profile, including lower TC and TG,^41,42^ and more consumption of a dietary pattern including saturated fats, red meat, sweets, soft drinks, and refined grains which were also identified in our unhealthy breakfast pattern may be associated with dyslipidemia.^40,41^ The healthy breakfast dietary pattern in the present study contains foods with higher fiber and nutrients. High fiber intake, especially soluble fiber, can increase fat excretion. Also, fiber found in whole grains and vegetables, as well as high-protein foods, can increase satiety and improve appetite control.^42,44,45^ Mono-unsaturated fatty acids (MUFAs) are the main source of fatty acids in the Mediterranean dietary pattern, and in our study, nuts, which are among the sources of MUFAs, were included in the healthy dietary pattern. It has been shown that intakes of MUFA may prevent the development of NAFLD by improving plasma lipid levels and reducing body fat accumulation. Thus, the intake of high fiber, MUFAs, and PUFAs in the healthy breakfast dietary pattern in our study might be involved in reducing TC in the studied subjects.^45^ On the other hand, the unhealthy breakfast pattern was more energy-dense and rich in saturated fat, trans fat, and added sugar. It has been demonstrated that these food items elevate risk factors of NAFLD.^42,46^ For example, saturated fatty acids cause mitochondrial dysfunction, which leads to the development and progression of NAFLD.^47^ In addition, excessive or inappropriate carbohydrate intake combined with insulin resistance leads to a glucose shunt in the liver, where it is converted either to free fatty acids or to glycogen via insulin-stimulated de novo lipogenesis. Motivating the production of free cholesterol leads to the accumulation of TG and cholesterol esters in the liver.^48^ Although HDL-C had no meaningful association with breakfast dietary patterns in our study, this may be because of some other factors. HDL-C tends to be less sensitive to short-term or meal-specific dietary exposures than other lipid fractions such as TC and TG. Moreover, HDL-C concentrations are heavily affected by non-dietary factors such as physical activity, smoking, adiposity, and hormonal status, which can attenuate associations in observational analyses. Dietary effects on HDL-related outcomes may be more pronounced for HDL function rather than HDL-C concentration per se, and chronic habitual diet quality may exert a greater influence on HDL-C than the pattern of a single meal. Together, these factors may explain the absence of a meaningful association between breakfast dietary patterns and circulating HDL-C in our study.

 According to the findings, a direct relationship was not found between the consumption of either breakfast patterns and serum FBS levels. Likewise, some other studies did not report a meaningful relationship between different dietary patterns and FBS in NAFLD or other diseases.^40,43,44,49,50^ On the other hand, in a study by Lesani et al.^38^ in Iranian adults, a higher consumption of the “oil, eggs, and grains” dietary pattern was directly related to serum FBS levels at breakfast. Greater adherence to the “bread, vegetables, and cheese” dietary pattern was associated with lower serum FBS levels.^38^ In our study, the second and third tertiles of the unhealthy breakfast dietary pattern also positively correlated with FBS. This finding suggests that if adherence to an unhealthy breakfast pattern increases among the study population, followed by a decrease in adherence to a healthy breakfast pattern, FBS levels may increase in the long term and elevate the risk of T2D in the study population. However, as expected, the mean of FBS was in the normal range due to the exclusion of subjects with diabetes from the study.

 According to the results, the mean serum levels of ALT and AST were higher than normal values (40 U/L and 35 U/L, respectively). No significant association was found between consuming a healthy breakfast dietary pattern and ALT and AST levels. Nevertheless, high consumption of an unhealthy breakfast dietary pattern in this study was positively correlated with increased ALT and AST. Similarly, in the study by Totunchi et al,^40^ a greater consumption of dietary patterns, including sweets, hydrogenated fat, red and processed meat, and soft drinks, had a significant direct relationship with ALT in Iranian adults with NAFLD. The unhealthy breakfast pattern in our study was rich in saturated fats, trans fats, and added sugars. It has been shown that high consumption of simple sugars causes the production of storage fats (TG) in the body, especially in liver cells.^46^ Also, high consumption of saturated and trans fats can be associated with increased liver enzymes by increasing the level of free radicals in liver cells and increasing the possibility of cell damage.^51,52^

 Obesity is one of the most important risk factors associated with NAFLD. The average BMI and WC of the individuals in our study were in the overweight and obesity ranges, respectively. The results indicated that the consumption of a healthy breakfast dietary pattern was correlated with lower BMI. On the contrary, the consumption of an unhealthy breakfast dietary pattern had a positive relationship with BMI and WC. In the study of Lesani et al.^38^ on Iranian adults, higher consumption of the breakfast pattern “oil, eggs and cereals” was directly associated with higher BMI. While the relationship between these two variables was inverse in individuals with higher adherence to the breakfast pattern of bread, vegetables, and cheese.^38^ The benefits of a healthy breakfast pattern on reducing obesity values can be explained by a low glycemic index, low energy density and protein (in low-fat dairy and nuts), and high fiber (in vegetables), and low-fat intake (especially animal fats) and its effect on reducing appetite and food intake. Whereas, the consumption of high-carbohydrate foods with a high glycemic index in an unhealthy dietary pattern causes a high glycemic response and increases the oxidation of carbohydrates to fats, leading to energy storage (increased fat storage).^53,54^ Our results also confirm that unhealthy breakfast dietary patterns may lead to the accumulation of body fat, especially visceral fat.

 In terms of disease severity, the odds of high-grade NAFLD decrease with consumption of healthy breakfast dietary patterns and increase with consumption of unhealthy breakfast dietary patterns. This finding is consistent with the results of a clinical trial conducted by Sedgi et al.^55^ on 110 patients with NAFLD in 2024 that demonstrated consuming canola oil instead of animal oil for 12 weeks improved hepatic steatosis in NAFLD patients. Also, the consumption of nuts and seeds in a healthy breakfast dietary pattern, which contains healthy fats, could lower LDL-C and increase HDL-C. This balance in fat levels reduces the risk of liver disease.^54^ The healthy breakfast pattern contained complex carbohydrates and higher protein. These macronutrients help stabilize blood sugar levels. This reduces insulin secretion, which in turn diminishes fat storage in the liver.^56,57^ Fibers in a healthy breakfast dietary pattern improve gut function. A healthy gut plays an important role in fat metabolism and detoxification.^58^ The healthy breakfast dietary pattern also includes more vitamins and minerals. These nutrients help support liver health and function and can reduce inflammation.^59^ Antioxidants and anti-inflammatory compounds in healthy dietary patterns can reduce inflammation in the liver, which is one of the factors that lead to NAFLD. On the other hand, in an unhealthy breakfast pattern, the consumption of simple sugars causes a sudden increase in blood sugar levels and subsequent high insulin secretion, which leads to more fat accumulation in the liver and can increase the development of NAFLD.^56^

 A possible explanation for the inconsistency of the findings with previous reports may be due to differences in metabolic characteristics of individuals, dietary assessment methods, dietary habits of different societies, and other lifestyle-related factors, as well as individual differences in the bioavailability of nutrients.

## Limitations and strengths

 It should be noted that the cross-sectional nature of this study does not allow for cause-and-effect conclusions. The self-reported dietary data, possible recall bias, relatively low KMO value, and use of the non-random convenience sampling method are other limitations of this study. Moreover, breakfast dietary patterns vary in different regions of the world and within a country based on geographical, cultural, social, and economic factors. Therefore, the results cannot be generalized to the entire population with NAFLD. However, our results open new hypotheses about the best choice for breakfast to lower NAFLD risks. To obtain more complete results regarding the relationship between breakfast dietary patterns and blood sugar, it is recommended that blood insulin levels, insulin resistance, and insulin sensitivity be considered in future studies, too. Also, we did not measure sleep duration, chronotype, stress, or socio-economic status, which can influence breakfast patterns; these should be measured in future studies.

## Implications

 The findings of this study can help nutrition and health policymakers design appropriate preventive and intervention programs, including the necessary educational programs and behavioral recommendations, e.g., increasing intake of whole grains, vegetables, and low-fat dairy at breakfast to reduce the risk of NAFLD and related chronic diseases, such as metabolic syndrome, diabetes, and cardiovascular diseases, in the community.

## Conclusion

 The findings identified two breakfast dietary patterns, including healthy and unhealthy breakfast dietary patterns, by factor analysis. The healthy breakfast dietary pattern was associated with decreased serum TC levels, overall obesity, and the chance of developing high-grade NAFLD. Moreover, the unhealthy breakfast dietary pattern was related to increased ALT and AST concentrations, overall and abdominal obesity, and the chance of developing high-grade NAFLD. These achievements may help inform the design of dietary interventions and educational programs for improving NAFLD. Further longitudinal and interventional studies are required to clarify the results.

## Competing Interests

 The authors have declared that no competing interests exist.

## Ethical Approval

 This study protocol was approved by the Ethics Committee of Tabriz University of Medical Sciences (Ethics Code: IR.TBZMED.REC.1402.750).

## Supplementary File

Table S1. Food groups used in the analysis of breakfast dietary patterns of adults
