# Supplementary material for: Breakfast Dietary Patterns and Their Associations with Metabolic Indicators and NAFLD Severity: A Cross-Sectional Study Among Iranian Adults
Source: Health Promot Perspect. 2026 Aug 21;16(2):253–63. doi: 10.34172/hpp.45534 (PMC13723381; doi:10.34172/hpp.45534)
Supplement: Supplementary file 1 [file hpp-16-253-s001.pdf]

Kianmehr et al, Health Promotion Perspectives 2026;16(2):253-S1
doi:10.34172/hpp.45534
https://hpp.tbzmed.ac.ir

Supplementary Table S1. Food groups used in the analysis of breakfast dietary patterns of adults with NAFLD

**Food Group**	**Food items**
Refined grains	White breads (lavash, baguettes), ice cream bread, starch, wheat flour, rice, pasta, noodles
Whole grains	dark breads (barbari, sangak, taftun), barley bread, wheat, sprouts, corn
Vegetables	Lettuce, spinach, greens, dark greens, parsley, watercress, coriander, dill, leek, mint, basil, grape leaves, beet leaves, onions, cucumbers, chives, radishes, turnips, rhubarb, artichokes, Eggplant, pumpkin, celery, pepper, bell pepper, beet, shallot, garlic, mushroom, okra, green peas, beans, green beans, carrots, pumpkin pudding
Potato	Potatoes
Tomato	Tomatoes, tomato juice, tomato paste, ketchup
Garlic	Garlic
Onion	Onion
Olive	Olive
Pickles	Pickles, types of pickling
Egg	Egg white, egg yolk
Fish	Fish, tuna
Poultry	Poultry
Processed meats	Sausages
Offal	Liver, lung, heart, giblets, chicken liver, gizzard, tripe, head, tongue, brain
Red meat	Beef, lamb, camel meat, hamburger
Legumes	Lentils, all kinds of beans, chickpeas, cobs, soybeans, mung beans, beans
Nuts	Chickpeas, walnuts, hazelnuts, almonds, pistachios, peanuts, cashews, sesame, seeds
Tea and coffee	Tea and coffee
Soft drinks and industrial juices	Industrial fruit juices with added sugar, soft drinks, syrups, malt beer
Fruits, natural juices	Apples, oranges, tangerines, dates, watermelons, cantaloupe, bananas, sweet lemons, apricots, grapes, unripe grape, cherries, pomegranates, strawberries, kiwis, grapefruits, persimmons, pears, peaches, Plums, nectarines, blackberries, parsnips, blueberries, figs, hawthorns and natural fruit juices
Dried fruit	Dried fruits and fruit leather
Low-fat dairy products	Low-fat milk, Low-fat cocoa milk, low-fat yogurt, low-fat cheese, pasteurized curd, buttermilk, Qarat
Full-fat dairy products	Full-fat milk, full-fat yogurt, full-fat cheese
Solid fats	Coconut, cream, cream cheese, chocolate cheese, pizza cheese
Liquid oils	Sunflower oil, corn oil, olive oil, Canola oil, rapeseed oil
Animal oil and butter	Cow oil, sheep oil, industrial butter, local butter, tail, milks fat
Mayonnaise	Types of mayonnaise and salad dressings

Sweets	Cake, cookie, candies, dumpling, gaz, sohaan, noghl, Danish, sugar cubes, sugar, chocolate, pastille, jelly, caramel cream, chocolate biscuit, all kinds of jams and compotes, biscuit with cream
